# Editorial: Drug Repurposing

**DOI:** 10.3389/fmed.2019.00154

**Published:** 2019-07-03

**Authors:** Pan Pantziarka, Nicolas André

**Affiliations:** ^1^Anticancer Fund, Brussels, Belgium; ^2^The George Pantziarka TP53 Trust, London, United Kingdom; ^3^Metronomics Global Health Initiative, Marseille, France; ^4^Department of Pediatric Hematology-Oncology, Assistance Publique Hôpitaux de Marseille, La Timone Hospital, Marseille, France; ^5^Centre de Recherche en Cancérologie de Marseille (CRCM, Marseille Cancer Research Centre), SMARTC, Inserm UMR1068, CNRS UMR7258, Aix-Marseille University UM105, Marseille, France

**Keywords:** drug repurposing, oncology, clinical cancer research, drug development, drug repositioning

Drug repurposing, also known as drug repositioning or reprofiling, seeks new medical treatments from among existing medications rather than through the development of de novo medicines. Although interest in drug repurposing has increased in recent years, it is part and parcel of normal commercial drug development, particularly in the field of oncology (1). The standard business model is to gain a license for a new drug in a particular cancer indication and then to seek to extend this to new cancers or settings (i.e., from second line to first line treatment). However, there are particular challenges associated with repurposing off-patent or generic drugs (2). These challenges are as much to do with economic incentives and social policy as they are scientific or medical. Repurposing then, as a specific topic, encompasses both scientific and socio-political issues, as reflected in the range of papers in this Research Topic.

On the scientific side a key area is candidate identification—analogous to drug discovery outside of repurposing—that is the identification of specific licensed drugs which may have anticancer properties in one or more cancer types. Chamaraux-Tran and Piegeler outline evidence for one particular drug, the local anesthetic lidocaine and indicate how this may influence the course of metastatic spread following cancer surgery. Drawing on a wide range of evidentiary sources, the paper highlights both the anti-metastatic properties of lidocaine, particularly those mediated by effects on immunity, and on the direct anticancer effects of the drug. At a time when interest in perioperative therapies is increasing (3) this narrative review is most timely.

In contrast, Andresen and Gjertsen, adopt a disease-centric approach to survey a range of possible repurposing candidates for the treatment of acute myeloid leukemia (AML). In this very wide-ranging review potential AML treatments are identified from existing licensed chemotherapy agents (e.g., cladribine, clofarabine, melphalan etc.) and non-cancer drugs (metformin, statins, mebendazole etc.). Particular attention is focused on a number of very promising agents which are the subjects of clinical investigation including valproic acid and the anti-malarial quinacrine.

Case reports are an important source of evidence to support further investigation for repurposing candidates. Berland et al. present here a case of refractory pediatric atypical teratoid rhabdoid tumor treated with a combination treatment based on a backbone of metronomic chemotherapy and including the non-steroidal anti-inflammatory drug celecoxib. The case underlies the potential of the metronomics approach, that is the combination of low-dose metronomic chemotherapy with repurposed non-cancer drugs (4). A second case report included in this Research Theme is by Cornelius et al., showing a sustained response in a case of recurrent and metastatic choroid plexus carcinoma. In this case, arising as part of a clinical trial of molecularly guided therapy (NCT01802567), treatment consisted of a combination of with a combination of sirolimus, thalidomide, sunitinib, and vorinostat. Both of these challenging cases featured the use of novel combination therapies, including repurposed non-cancer drugs, leading to dramatic improvements in outcomes with manageable toxicity for pediatric patients.

Verschuur et al. report results from a metronomics clinical phase II trial (NCT01285817) assessing the combination of celecoxib, vinblastine, alternating oral cyclophosphamide and oral methotrexate in pediatric brain tumor patients. While a number of patients with low grade gliomas (LGG) showed positive responses to the treatment, responses were lacking in patients suffering from other brain tumor subtypes (e.g., ependymoma, medulloblastoma etc.). Interestingly among the positive responses were LGG patients who had previously been treated with vinblastine or progressed while on vinblastine.

Hernandez et al. outline a number of the financial disincentives which can stand in the way of developing repurposed therapies. While there are clear incentives for investing in the further development of a licensed drug when it is protected by intellectual property law, the situation is very different when the drug has moved beyond the commercial patent-protection period. This situation leaves a “funding gap” which may act as an obstacle to successful repurposing and therefore requires alternative funding strategies some of which are outlined in the paper.

Finally, Pantziarka et al. make the case for the inclusion of repurposed drugs in precision oncology trials and clinical practice. Pointing out that by definition precision oncology is about drug repurposing, the authors make the case that precision oncology, when delivered as part of a trial or within the context of clinical practice, should maximize the number of “actionable targets” by considering all licensed medications rather than restricting to existing cancer treatments or only targeted agents.

## Author Contributions

All authors listed have made a substantial, direct and intellectual contribution to the work, and approved it for publication.

### Conflict of Interest Statement

The authors declare that the research was conducted in the absence of any commercial or financial relationships that could be construed as a potential conflict of interest.
